# Clinical response of tuberculosis patients, a prospective cohort study

**DOI:** 10.1371/journal.pone.0190207

**Published:** 2018-01-02

**Authors:** Berhanu Elfu Feleke, Getu Degu Alene, Teferi Elfu Feleke, Yalmezerf Motebaynore, Fantahun Biadglegne

**Affiliations:** 1 Department of Epidemiology and Biostatistics, University of Bahir Dar, Bahir Dar, Ethiopian; 2 Department of Epidemiology and Biostatistics, University of Bahir Dar, Bahir Dar, Ethiopian; 3 Department of Pediatrics, University of St. Paul, Addis Ababa, Ethiopian; 4 Department of Internal Medicine, University of Bahir Dar, Bahir Dar, Ethiopian; 5 Department of Microbiology, University of Bahir Dar, Bahir Dar, Ethiopian; Indian Institute of Technology Delhi, INDIA

## Abstract

Clinical response means a response to drug intake that can be detected and appreciated by a change in signs and symptoms. The objectives of this study were to assess time to clinical response, the incidence density for clinical response and determinants of clinical response of tuberculosis (TB) patients in the intensive phases of TB treatment. Prospective cohort study design was implemented. The target population for this study was all patients following the directly observed therapy. Baseline data has been collected during the start of the directly observed TB treatment strategy. We have been collected updated data after the seven days of the baseline data collection, then after every seven days updated data has been collected from each pulmonary and extra pulmonary TB patients. Kaplan Meier curve was used to estimate time to clinical response. Incidence density using person days was used to estimate incidence of clinical response. Cox proportional hazard model was used to identify the predictors of clinical responses. A total of 1608 TB patients were included with a response rate at 99.5%. The mean age of the respondents was 24.5 years [standard deviation (SD) 14.34 years]. The incidence density for clinical response was 1429/38529 person days. One fourth of the TB patients showed clinical response at day 14, 25% of at day 21 and 75% o at day 31. Predictors of clinical response for TB patients includes: age (AHR 1.007 [95% CI 1.003–1.011]), type of TB (AOR 2.3[95% CI 2.04–2.59]), Previous history of TB (AHR 0.18 [95% CI 0.11–0 .30]), Intestinal parasitic infection (AOR 0.22[95% CI 0.19–0.26]), hemoglobin (AOR 2.35 [95% CI 2.18–2.54]), weight gain (AOR 1.11 [95% CI 1.05–1.17]), Micronutrient supplementation (AOR 9.71 [95% CI 8.28–11.38]), male sex (AOR 0.87 [95% CI 0.79–0.97]).The clinical responses for extra-pulmonary TB patients were slower than pulmonary TB. Deworming and micronutrient supplementation should be considered as the additional TB treatment strategy for TB patients.

## Introduction

Clinical response means a response to drug intake that can be detected and appreciated by a change in signs and symptoms [1]. Tuberculosis is a chronic infectious disease caused by mycobacterium species and broadly classified as pulmonary tuberculosis (PTB) and extra pulmonary tuberculosis (EPTB). Patients with PTB manifest with ‘productive cough mixed with blood, anorexia, cachexia, low grade fever, night sweats, fatigue, dull chest pain and malaise’[2]. However, the manifestation of EPTB depend on the organ affected by the disease: tuberculosis lymphadenitis(TBLN) manifest with slowly developing and painless lymphadenophaty, leading to matting followed by drainage of pus; TB affecting the lining of the lung manifest with chest pain exacerbated by breathing in(inspiration), on and off type of cough, dyspnea; bones and joints TB manifest with numbness and/or swelling usually pus, myalgia, weakness of the extremity, pain during joint movement; intestinal TB manifest with anorexia and weight loss, abdominal discomfort, diarrhea, bloating, organomegally, ascite; TB affecting the central nervous system manifest with projectile vomiting, fever, headache, neck stiffness and delirium[2, 3].

Globally one-third of the world population was infected with TB, every year TB affects 10 million people [2–4], every 15 seconds one person die from TB comprising more than 2 million death annually [2–6].

The Africa continent consists of 28% of the world’s TB cases. In Ethiopia TB is the leading cause of morbidity, the incidence of all forms of TB in Ethiopia was 379/100000 population, which is second from the African continent [7–9]. Tuberculosis was also the leading cause of hospital admission and the second cause of death affecting more than 76000 people annually [2, 3, 10–14].

Literature across the globe revealed that clinical response during the directly observed therapy was determined by Human Immunodeficiency Virus (HIV) infection, older age, previous treatment, initial body mass index, alcoholism, diabetes, renal disease, late initial treatment, intestinal parasitic infection, COPD(chronic obstructive pulmonary disorder), treatment category, organ affected, micronutrient supplementation, diagnostic uncertainty[15–29].

A study that addresses the clinical response of TB patients in the resource limited setting including Ethiopia is limited. Slow clinical response leads to prolonged infectiousness, increasing the transmission of TB to the community which could be a barrier to the TB control and prevention program. In addition, it has impact on the duration of TB treatment, work burden to the health institution, occurrence and, increasing the burden of multidrug resistance tuberculosis (MDR-TB) in the country, recurrence that affects the uptake of TB therapy [2, 30–32]. Therefore, this study aimed to assess time to clinical response, the incidence density of clinical response and determinants of clinical response of TB patients in the intensive phase of TB treatment.

## Methods and materials

Prospective cohort study design was implemented. The clinical response of PTB patients were compared with the clinical response of EPTB patients. Both groups were followed for the entire period of intensive phase. This study was conducted in Amhara region, one of the eleven regions in Ethiopia. The study was conducted in health centers because most of the directly observed therapies were followed in the health centers. The data were collected from July 2016-May 2017. The target population for this study was all TB patients following the directly observed therapy. Patients simultaneously attached with PTB and EPTB were excluded to avoid misclassification bias. The sample size was calculated using Epi-info software version 7 using the assumption of 95% CI, power of 90%, 1:1 ratio of EPTB to PTB patients, none response rate of 10% and design effect of 2 gives 808 PTB patients and 808 EPTB patients. A multistage sampling technique was used. A simple random sampling was used to select the zones. First, representative 3 zones were selected from the eleven zones in the region. A stratified sampling technique was used to select health centers from the 3 zones. From each health center, TB patients were selected using systematic random sampling technique during the start of DOTS. Initially, baseline data were collected during the start of DOTS regimen using interview technique. The data were collected by 30 nurses and supervised by 9 health officers. The data were collected in continues phase every week until they show the clinical response. Digital weight scale was used to measure the weight of each patient and measured to the nearest 0.1 kilogram[33]. The blood and stool samples were collected by 6 first degree holder medical laboratory technologists and supervised by three medical microbiologists. A one-gram stool sample was collected from each patient’s in 10 ml SAF (sodium acetate- acetic acid-formalin solution). A concentration technique was used. The stool sample was well mixed and filtered using a funnel with gauze then centrifuged for one minute at 2000 RPM (revolution per minute) and the supernatant was discarded. Following this 7 ML (Milliliter) of normal saline was added, mixed with a wooden stick, 3 ML ether was added and mixed well then centrifuged for 5 minutes at 2000 RPM. Finally, the supernatant was discarded and the whole sediment was examined for parasite[34]. A 5 ML blood sample was collected from patients following the standard operational procedures to measure the hemoglobin level of patients using Mindray hematology analyzer. Data were entered into the computer using Epi-info software and transferred to STATA for analysis. Kaplan Meier curve was used to estimate time to the clinical response. Incidence density using person days and person weeks were used to estimate the incidence of clinical response[35]. Parametric Cox proportional hazard model (Weibull) was used to identify the predictors of clinical responses. Adjusted Hazard risk with their 95% CI was used to identify the predictors of clinical response. To increase the quality of collected data first questionnaire was prepared in English and translated to Amharic language (local language), Pretest was conducted on 5% of the samples, training was given to the data collectors and supervisors, data cleaning was performed to decrease missing data and outlier, close supervision was conducted by supervisors and investigators. Our event was the clinical response. The patient was followed until the manifestations of TB disappeared. Patients lost from the study or who completed the study period without clinical response was considered as a censored observation. We declare clinical response when the initial sign and symptoms disappeared.

This study was ethically approved by Bahir Dar University, College of medicine and health sciences ethical committee. Support letter was obtained from Amhara national regional state health bureau. Legal permission was obtained from the respective authorities. Written informed consent was obtained from each study participants. The right to withdraw from the study at any point was respected for each study participants. Patients with the intestinal parasite or with low hemoglobin concentration were referred for further managements.

## Results

A total of 1608 TB patients were included giving the response rate of 99.5%. The mean age of the respondents was at 24.5 years [standard deviation (SD) 14.34 years].

### Profile of pulmonary tuberculosis patients

**Fig 1:**
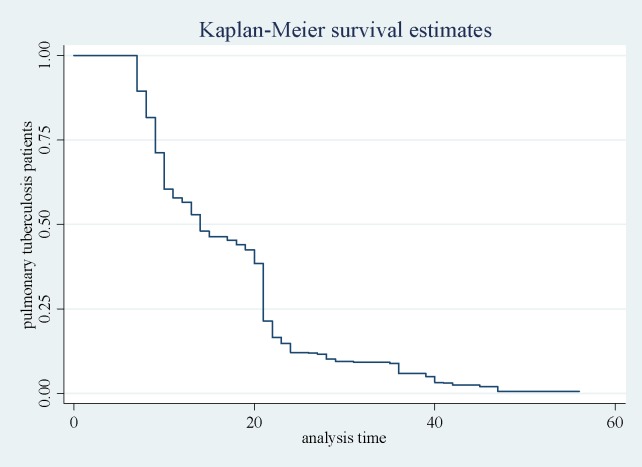
Kaplan-Meir and predicted survival plot for pulmonary tuberculosis patients.

Totally 804 PTB patients were followed during the entire period of the study. The mean age of the patients under this category was 20.86 years (SD = 11.53 years). The total time at risk for this category was 13582 days. The incidence density of the clinical response for PTB patients was at 793/13582 person days. Most of the PTB patients (97.6%) showed the clinical response at the 56 days of intensive phase, 25%, 50% and 75% of the cases showed clinical response at day 9, 14 and 21, respectively (Fig 1).

Half of the study participants were female, 62% of the study populations were from the rural area and 70% of the study participants were less than 20 years old (Table 1).

**Table 1:** Socio-demographic characteristics of pulmonary tuberculosis patients (n = 804).

SN	Population profile		Frequency	Percentage
1.	Sex	Male	405	50.37
		Female	399	49.63
2.	Residence	Urban	304	37.81
		Rural	500	62.19
3.	Religion	Orthodox	777	96.64
		Muslim	20	2.49
		Protestant	7	0.87
4.	Ethnicity	Agaw	60	7.46
		Amhara	702	87.31
		Oromo	9	1.12
		Tigray	23	2.86
		Others	10	1.24
5.	Educational status	Literate	594	73.88
		Illiterate	210	26.12
6.	Marital status	Single	437	54.35
		Married	359	44.65
		Divorced	7	0.87
		Widowed	1	0.12
7.	Age	0–20 years	569	70.77
		21–40 years	184	22.89
		41–60 years	27	3.36
		60 + years	24	2.99

#### Population profile of extra-pulmonary tuberculosis patients

**Fig 2:**
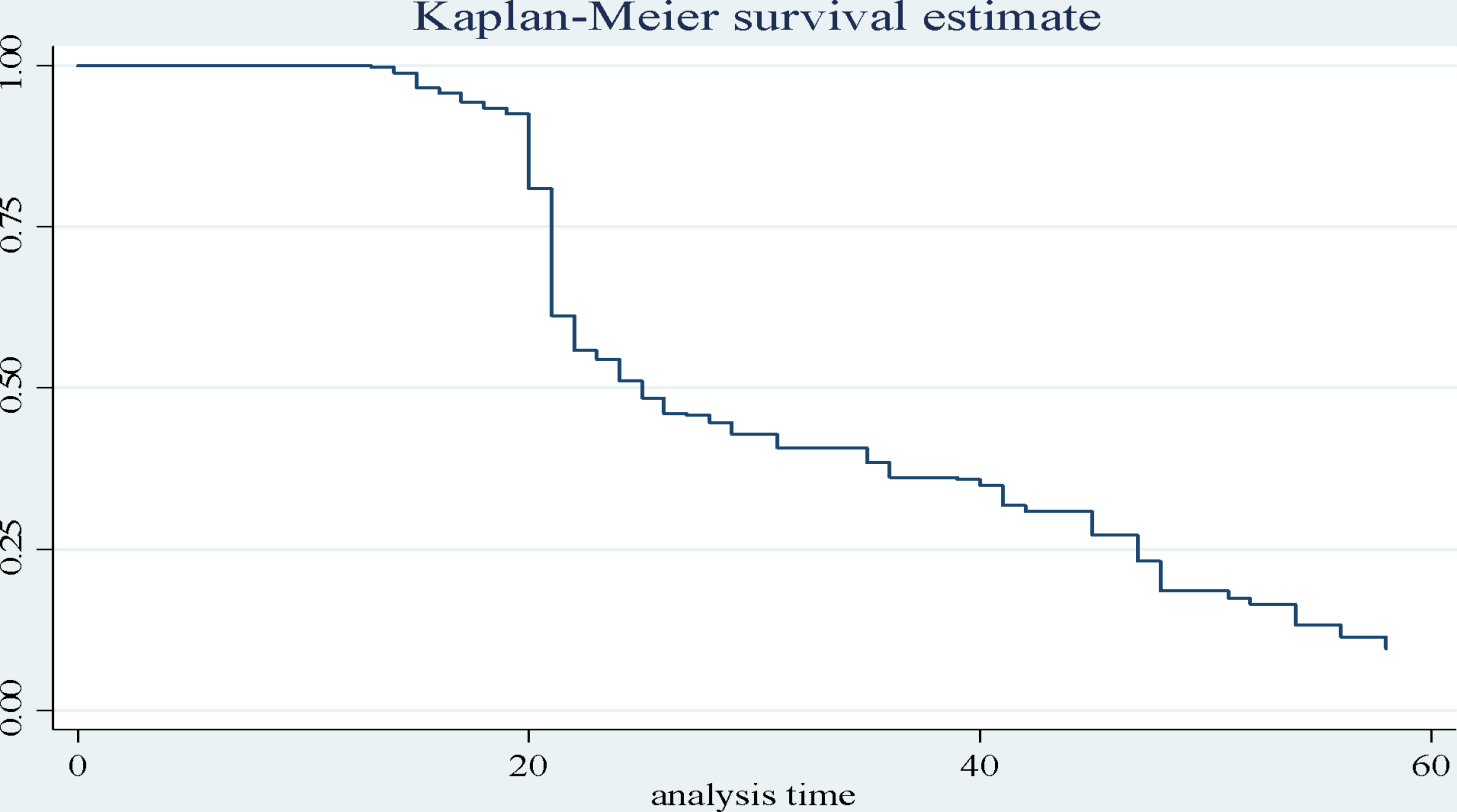
Kaplan Meier survival estimate for extra-pulmonary tuberculosis patients.

A total of 804 EPTB patients were included. The mean age of the respondents was 28.14 years (SD 15.88 years). The incidence density for clinical response among EPTB patients was 636/24946 person days (Fig 2).

Female constituted 50% of the EPTB patients, 53.48% of the study participants were from the rural area, and 50% of the study participants were within the age group of 21–40 years (Table 2).

**Table 2:** Socio-demographic characteristics of extra-pulmonary tuberculosis patients (n = 804).

SN	Population profile		Frequency	Percentage
1.	Sex	Male	419	52.11
		Female	385	47.89
2.	Residence	Urban	374	46.52
		Rural	430	53.48
3.	Religion	Orthodox	776	96.52
		Muslim	25	3.11
		Protestant	3	0.37
4.	Ethnicity	Agaw	50	6.22
		Amhara	712	88.56
		Oromo	14	1.74
		Tigray	21	2.61
		Others	7	0.87
5.	Educational status	Literate	618	76.87
		Illiterate	186	23.13
6.	Marital status	Single	212	26.37
		Married	581	72.26
		Divorced	10	1.24
		Widowed	1	0.12
7.	Age	0–20 years	293	36.44
		21–40 years	402	50
		41–60 years	27	3.36
		60 + years	82	10.2

### Clinical response of tuberculosis patients

**Fig 3:**
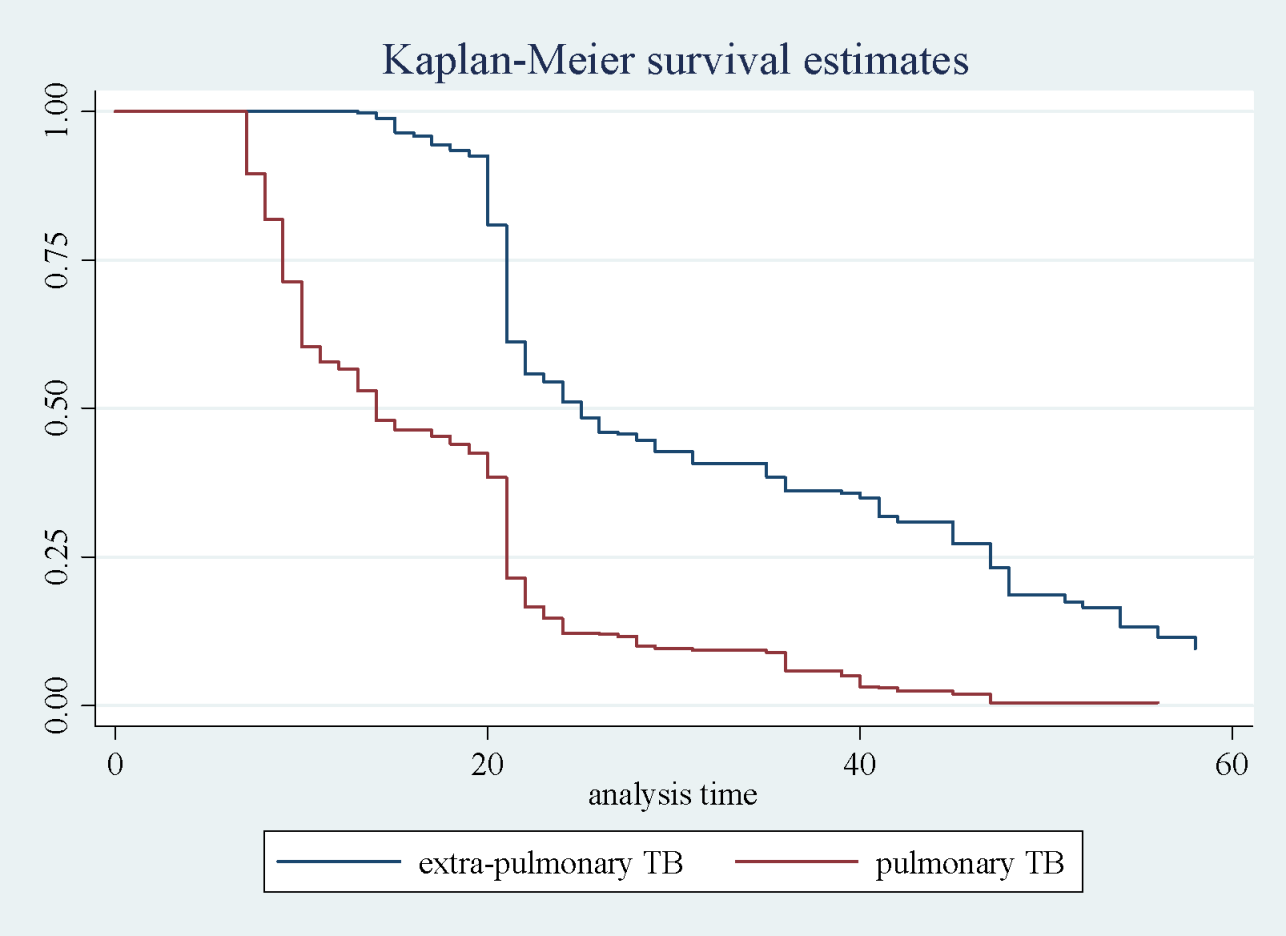
Kaplan-Meier curve for pulmonary tuberculosis and extra-pulmonary tuberculosis patients.

The incidence density for clinical response was 1429/38529 person days. One fourth, 50% and 75% of the TB patients indicated the clinical response at day 14, 21 and 31 respectively. The clinical response was slow for EPTB patients compared to PTB patients (Fig 3).

**Fig 4:**
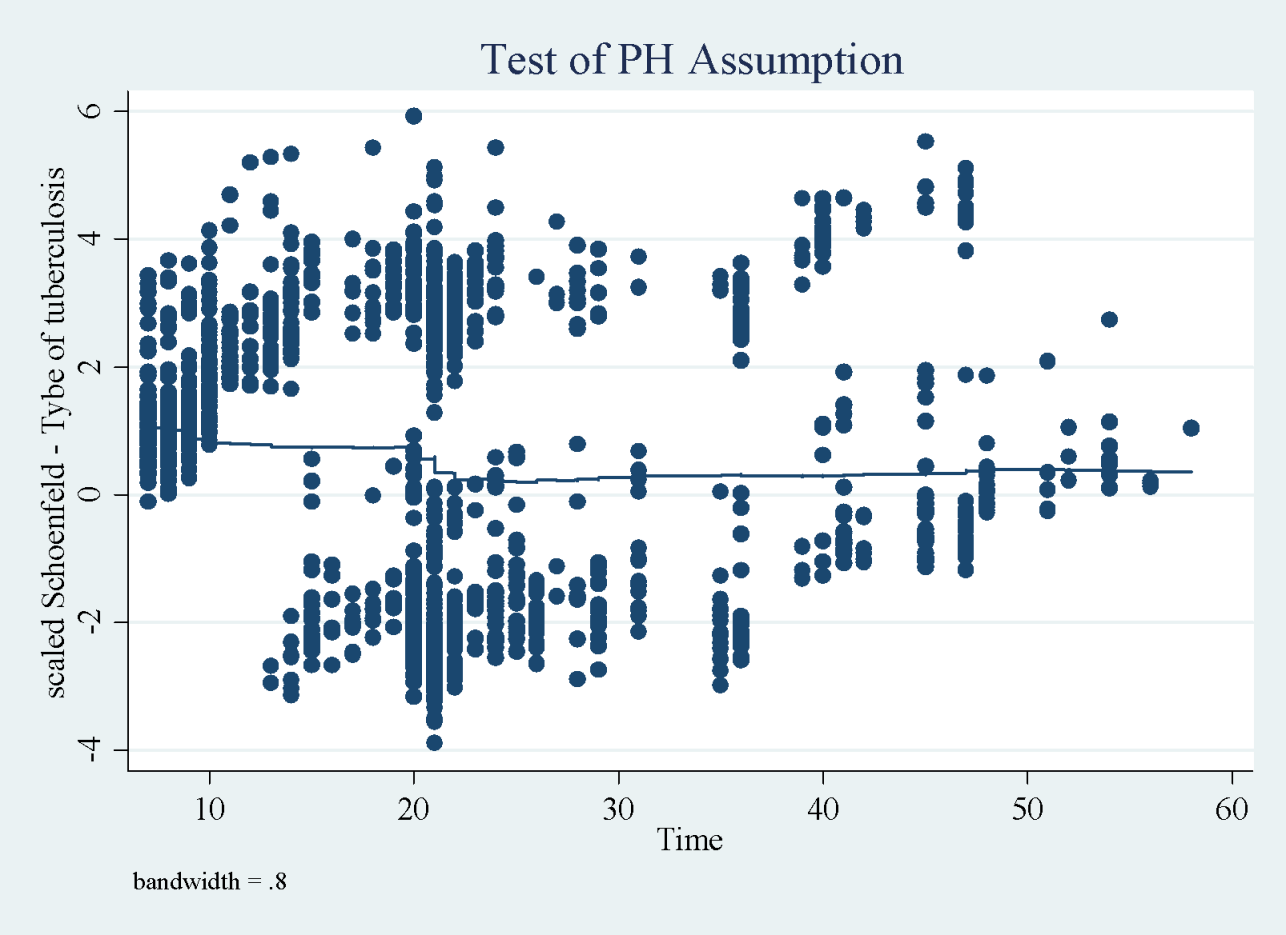
schoenfeld residuals plot for pulmonary and extra-pulmonary tuberculosis patients.

The proportional hazard assumption was checked using schoenfeld residuals graph and no pattern was observed meaning that the assumption was fulfilled (Fig 4).

The predictors of clinical response were age, type of TB, previous history of TB, intestinal parasite, hemoglobin concentration, weight gain, micronutrient supplementation, and sex (Table 3).

**Table 3:** Predictors of clinical response in TB patients.

Variables	CHR (95% CI)	AHR (95% CI)	p-value
Age	0.984 (0.980–0.988)	1.007(1.003–1.011)	0.001
Type of tuberculosis	3.7 (3.32–4.13)	2.3(2.04–2.59)	<0.01
Previous history of tuberculosis	0.096 (0.058–0.166)	0.18(0.11–0 .30)	<0.01
Intestinal parasite infection	0.12 (0.11–0.14)	0.22(0.19–0.26)	<0.01
Hemoglobin	2.49 (2.34–2.65)	2.35 (2.18–2.54)	<0.01
Other chronic illness	1.24 (1.07–1.43)	1.01 (0.87–1.17)	0.88
Weight gain	1.29 (1.24–1.35)	1.11 (1.05–1.17)	<0.01
Micronutrient supplementation	8.73 (7.62–10.01)	9.71 (8.28–11.38)	<0.01
Sex	0.99 (0.89–1.11)	0.87 (0.79–0.97)	0.01
Residence	1.03(0.93–1.15)	1.02 (0.92–1.14)	0.75

In this study, the clinical response is lower in children, i.e. as the age of the patient increase by one year, the clinical response increase by 1% (AHR 1.007 [95% CI 1.003–1.011]). The clinical response of PTB patients was 2.3 times higher than the clinical response of EPTB patients (AOR 2.3[95% CI 2.04–2.59]). In addition the clinical response of TB patients with previous history of TB was 82% lower than the TB patients with no history of previous TB (AHR 0.18[95% CI 0.11–0 .30]). In this study, patients with the intestinal parasites showed lower clinical response compared to patients without intestinal parasites (AOR 0.22[95% CI 0.19–0.26]). The clinical response is higher in patient with higher hemoglobin concentration; increasing the hemoglobin concentration of TB patient by 1 g/dl increases the clinical response by 2.35 folds higher (AOR 2.35 [95% CI 2.18–2.54]). In this study, weight gain during the intensive phase of DOTS regimen increases the clinical response; per 1 kg increase in the weight of the patient, 11% increment in the clinical response will be obtained (AOR 1.11 [95% CI 1.05–1.17]). In our study, we also observed that micronutrient supplementation during DOTS increases the clinical response by 9.71 folds higher (AOR 9.71 [95% CI 8.28–11.38]). In this study, we noted that females patients had higher clinical response compared to males patients (AOR 0.87 [95% CI 0.79–0.97]).

## Discussion

In this study, the incidence density for clinical response among TB patients was 1429/38529 person days (26/100 person week), i.e. if we follow 100 TB patients for a week during the intensive phase of DOTS, 26 patients show the clinical response at the end of the week.

In this study, the clinical response in PTB patients was 2.3 times higher than the clinical response in EPTB patients (AOR 2.3[95% CI 2.04–2.59]). This research finding agrees with the findings from South Africa [15, 22, 23], Addis Ababa [29]. This might be due to the diagnostic uncertainty of EPTB patients [36]. In this study, we observed that the clinical response of TB patients with previous history of TB was 82% lower than the TB patients with no history of previous TB. This finding is inconsistent with the findings from south Africa [15], India [18]. This is due to the reason that TB patients in the retreatment category had poor treatment outcome than the other categories, because patients in the retreatment category usually contains mycobacterium species resistant to the first line anti-TB drugs and patients didn’t show the response even if they took their drug properly [37–39]. In this study, the clinical response in TB patients with the intestinal parasites was 78% lower compared to the clinical response in TB patient free from intestinal parasites, which is consistent with the finding from Brazil [24]. This might be due to the effect of intestinal parasites on the immune system of the host[40]. The humoral immune response at the result of intestinal parasites favors severity of mycobacterium infection and poor treatment response to chemotherapy [41, 42].

In this study, increasing the hemoglobin concentration of TB patients by 1 g/dl increased the clinical response by 2.35 folds higher. This finding is in agreement with finding from Tanzania [43], indicating anemia increased the severity of TB infection and resulted also in delayed response to anti TB therapy [44]. In our study, weight gain during the intensive phase of DOTS regimen increased the clinical response; per 1 kg increase in the weight of the patient, 11% increment in the clinical response will be obtained. This finding agrees with findings from Ethiopia [17, 29], India [18]. This is due to the good nutritional support of TB patient in this category meaning that they are in good progress [44, 45].

Micronutrient supplementation during DOTS increases the clinical response by 9.71 folds higher. This is comparable with findings from Iran [16]. This might be due to the effect of micronutrient on the cell mediated immunity, so, micronutrients play the great role in defense against infectious agents [46, 47]. In our study, we observed that the clinical response in female TB patients was 13% higher than male TB patients. This finding agrees with South Africa [22, 23], Hong Kong [28] findings. But disagree with the finding from India [27]. This is due to the reason that lifestyle modification and adherence to health professionals advice is higher in female patients in the study area [48]. In this study, clinical response is lower in children, as the age of the patient increase by one year, the clinical response increase by 1%. This finding disagrees with the findings from previous scholars [15, 22]. This is due to the reason that in the study area as the age of the individual gets higher the likely hood of engaging in daily exercise is higher which finally enhance the immunity to fight chronic infection[49]. In our study, the presence of other chronic illness, the residence of the patient has no effect on the clinical response of TB patients.

The limitation of this study might be the failure to obtain immunological markers during the update or longitudinal data collection phase, but since the main objective of this study was to obtain the clinical response it will not have a major effect on the study outcome.

## Conclusion

Clinical response of PTB patients was faster than EPTB patients. The median time of clinical response for tuberculosis patients was 21 days. Clinical response of tuberculosis patients was determined by age, previous history of TB, intestinal parasite, hemoglobin concentration, weight gain, micronutrient supplementation, and sex.

## Recommendation

Every tuberculosis patients must be screened for intestinal parasitic infection and treated well during the DOTS regimen. Every tuberculosis patients must be supplied with Micronutrients during DOTS.

## Supporting information

S1 File The data collection tool (Questionnaire for the study). (DOCX)

S2 File Stata file of the raw data. (DTA)
