# Supplementary material for: Clinical response of tuberculosis patients, a prospective cohort study
Source: PLoS One. 2018 Jan 2;13(1):e0190207. doi: 10.1371/journal.pone.0190207 (PMC5749762; doi:10.1371/journal.pone.0190207)
Supplement: S1 File — (DOCX) [file pone.0190207.s001.docx]

## ANNEX I Consent Form for Participation in a Research Study

**Title of Study: *Survival to Clinical response of tuberculosis patients, a prospective cohort study***

**Description of the research**

You are invited to participate in a research study conducted by the university of Bahir dar CMHS staff. The purpose of this research is to estimate time to clinical response and their determinants

**Risks and discomforts**

There are no known risks associated with this research.

**Potential benefits**

Patients with anemia or intestinal parasitic infection will be diagnosed using standardized procedures and advised intervention mechanism to avert their risk. This research givers important clues in averting MDR TB, recurrences and decreasing the severity of tuberculosis.

**Protection of confidentiality**

Name will not be written to the questioners and the confidentiality of the data will be kept at all stages

**Voluntary participation**

Your participation in this research study is voluntary. You may choose not to participate and you may withdraw your consent to participate at any time. You will not be penalized in any way should you decide not to participate or to withdraw from this study.

**Contact information**

If you have any questions or concerns about this study or if any problems arise please contact us using……,

**Consent**

**I have read this consent form and have been given the opportunity to ask questions. I give my consent to participate in this study.**

Participant’s signature_______________________________ Date:_________________

A copy of this consent form should be given to you.

***Baseline record***

1. Date of interview (DD/MM/YYYY)_______ ____________ __________________
2. Date of birth (DD/MM/YYYY)__________ __________ _______________________
3. Sex ______________
4. Educational status

Illiterate

Informal education

Formal education

1. Marital status

Single

Married

Divorced

Widowed

1. residence

Urban Rural

1. **Occupation**

Farmer

Merchants

Private employee

Pitty trader

House wife

Students

Unemployed

Retired

Government employee

NGO workers

Daily laborer

Others please specify

1. **Ethnicity**

Amhara

Agaw

Orormo

Tigray

Others please specify _________________________________

1. **Religion**

Orthodox

Muslim

Catholic

Protestant

1. **Type of tuberculosis**

Pulmonary tuberculosis

Extra pulmonary tuberculosis

1. If pulmonary tuberculosis, what was the smear result

Smear positive

Smear negative

1. If extra pulmonary tuberculosis mention the affected site _____________
2. Did the patient had previous history of tuberculosis

Yes

No

1. What are the specific manifestation of the case

- __________________________________________
- __________________________________________
- __________________________________________
- _________________________________________
- _________________________________________
- ________________________________________
- ________________________________________
- _______________________________________
- _______________________________________

1. Treatment category __________________
2. Did the patient have any known chronic illness

Yes

No

1. If yes, please mention the disease ------------------------------
2. Did the patient know his/her Serostatus?

Yes

No

1. If yes, what was the result

Negative

Positive

1. Length/height in centimeter ______________________
2. Weight in kilogram _____________________________
3. Hemoglobin concentration ________________________________
4. *Stool results* ______________________________________
5. Average monthly income ____________________

**Continues data collection**

**Visit one (first week)**

1. Date of interview (DD/MM/YYYY)___________ __________ __________
2. Weight (in kg)_________________
3. Alcohol intake (yes/no)
4. Micro nutrient supplementation (yes/no)______________________
5. Drug compliance ____________________
6. From the baseline record what are the sign and symptom disappeared (include also the date)

- _____________________________________
- ______________________________________
- _______________________________________
- _______________________________________
- _______________________________________
- ________________________________________

1. From the baseline record what manifestation still exist
   - _____________________________________
   - ______________________________________
   - _______________________________________
   - _______________________________________
   - _______________________________________
   - ________________________________________

**Continues data collection**

**Visit two (second week)**

1. Date of interview (DD/MM/YYYY)___________ __________ __________
2. Weight (in kg)_________________
3. Alcohol intake (yes/no)
4. Micro nutrient supplementation (yes/no)______________________
5. Drug compliance ____________________
6. From the baseline record what are the sign and symptom disappeared (include also the date)

- _____________________________________
- ______________________________________
- _______________________________________
- _______________________________________
- _______________________________________
- ________________________________________

1. From the baseline record what manifestation still exist
   - _____________________________________
   - ______________________________________
   - _______________________________________
   - _______________________________________
   - _______________________________________
   - ________________________________________

**Continues data collection**

**Visit three (third week)**

1. Date of interview (DD/MM/YYYY)___________ __________ __________
2. Weight (in kg)_________________
3. Alcohol intake (yes/no)
4. Micro nutrient supplementation (yes/no)______________________
5. Drug compliance ____________________
6. From the baseline record what are the sign and symptom disappeared (include also the date)

- _____________________________________
- ______________________________________
- _______________________________________
- _______________________________________
- _______________________________________
- ________________________________________

1. From the baseline record what manifestation still exist
   - _____________________________________
   - ______________________________________
   - _______________________________________
   - _______________________________________
   - _______________________________________
   - ________________________________________

**Continues data collection**

**Visit four (fourth week)**

1. Date of interview (DD/MM/YYYY)___________ __________ __________
2. Weight (in kg)_________________
3. Alcohol intake (yes/no)
4. Micro nutrient supplementation (yes/no)______________________
5. Drug compliance ____________________
6. From the baseline record what are the sign and symptom disappeared (include also the date)

- _____________________________________
- ______________________________________
- _______________________________________
- _______________________________________
- _______________________________________
- ________________________________________

1. From the baseline record what manifestation still exist
   - _____________________________________
   - ______________________________________
   - _______________________________________
   - _______________________________________
   - _______________________________________
   - ________________________________________

**Continues data collection**

**Visit five (fifth week)**

1. Date of interview (DD/MM/YYYY)___________ __________ __________
2. Weight (in kg)_________________
3. Alcohol intake (yes/no)
4. Micro nutrient supplementation (yes/no)______________________
5. Drug compliance ____________________
6. From the baseline record what are the sign and symptom disappeared (include also the date)

- _____________________________________
- ______________________________________
- _______________________________________
- _______________________________________
- _______________________________________
- ________________________________________

1. From the baseline record what manifestation still exist
   - _____________________________________
   - ______________________________________
   - _______________________________________
   - _______________________________________
   - _______________________________________
   - ________________________________________

**Continues data collection**

**Visit six (sixth week)**

1. Date of interview (DD/MM/YYYY)___________ __________ __________
2. Weight (in kg)_________________
3. Alcohol intake (yes/no)
4. Micro nutrient supplementation (yes/no)______________________
5. Drug compliance ____________________
6. From the baseline record what are the sign and symptom disappeared (include also the date)

- _____________________________________
- ______________________________________
- _______________________________________
- _______________________________________
- _______________________________________
- ________________________________________

1. From the baseline record what manifestation still exist
   - _____________________________________
   - ______________________________________
   - _______________________________________
   - _______________________________________
   - _______________________________________
   - ________________________________________

**Continues data collection**

**Visit seven (seventh week)**

1. Date of interview (DD/MM/YYYY)___________ __________ __________
2. Weight (in kg)_________________
3. Alcohol intake (yes/no)
4. Micro nutrient supplementation (yes/no)______________________
5. Drug compliance ____________________
6. From the baseline record what are the sign and symptom disappeared (include also the date)

- _____________________________________
- ______________________________________
- _______________________________________
- _______________________________________
- _______________________________________
- ________________________________________

1. From the baseline record what manifestation still exist
   - _____________________________________
   - ______________________________________
   - _______________________________________
   - _______________________________________
   - _______________________________________
   - ________________________________________

**Continues data collection**

**Visit eight (eighth week)**

1. Date of interview (DD/MM/YYYY)___________ __________ __________
2. Weight (in kg)_________________
3. Alcohol intake (yes/no)
4. Micro nutrient supplementation (yes/no)______________________
5. Drug compliance ____________________
6. From the baseline record what are the sign and symptom disappeared (include also the date)

- _____________________________________
- ______________________________________
- _______________________________________
- _______________________________________
- _______________________________________
- ________________________________________

1. From the baseline record what manifestation still exist
   - _____________________________________
   - ______________________________________
   - _______________________________________
   - _______________________________________
   - _______________________________________
   - ________________________________________
